# Assessment of the Exposure of People to Questing Ticks Carrying Agents of Zoonoses in Aosta Valley, Italy

**DOI:** 10.3390/vetsci6010028

**Published:** 2019-03-17

**Authors:** Ilary Millet, Marco Ragionieri, Laura Tomassone, Claudio Trentin, Alessandro Mannelli

**Affiliations:** 1 Department of Veterinary Sciences, University of Turin, 10095 Grugliasco, Italy; laura.tomassone@unito.it; 2 Dipartimento di Prevenzione, SC Sanità animale, Azienda USL della Valle d’Aosta, 11020 Quart, Italy; mragionieri@ausl.vda.it (M.R.); ctrentin@ausl.vda.it (C.T.)

**Keywords:** risk analysis, *Ixodes ricinus*, *Borrelia burgdorferi* s.l., *Rickettsia* spp., ticks, zoonoses, Italy

## Abstract

We estimated the probability of exposure of people to questing ticks, infected with bacterial agents of the tick—borne zoonoses—in Aosta Valley, western Alps, Italy. We collected ticks by dragging, and from collectors’ clothes in three hiking trails, which were divided into an internal path, with short vegetation, and an external part with taller grass. Dragging yielded 285 *Ixodes ricinus* nymphs and 31 adults, and two *Dermacentor marginatus* adults. Eleven *I. ricinus* nymphs and 9 adults were collected from collectors’ clothes. *Borrelia burgdorferi* s.l. was identified by PCR in 12 out of 30 *I. ricinus* nymphs (prevalence = 40.0%, 95% confidence interval = 22.5, 57.5). The prevalence of infection by *Rickettsia* spp. was 13.3% (95% CI = 1.2, 25.5). The probability of encountering at least one questing *I. ricinus* infected by each bacterial agent (probability of exposure, *E*) in 100 m^2^ was obtained by combining the number of collected nymphs, the prevalence of infection by each bacterial agent, the frequency of passage by visitors, and the probability of tick attachment to people. The mean number of nymphs collected by dragging was greatest in the internal part of hiking trails (mean = 7.9). Conversely, *E* was greater in the external part (up to 0.14 for *B. burgdorferi* s.l., and 0.07 for *Rickettsia* spp.), due to a greater probability of tick attachment to people in relatively tall vegetation.

## 1. Introduction

The hard tick *Ixodes ricinus* is the vector of zoonotic viruses, bacteria, and protozoa, across a wide geographic range, from southern Spain, to northern Scandinavia [1,2,3]. In recent decades, *I. ricinus* and transmitted agents have been reported in mountains in northwestern Italy, at altitudes greater than 1000–1200 m above the sea level (a.s.l.), which were previously considered as the maximum altitudinal limits of the tick’s geographic range [4,5,6,7,8,9,10,11]. In Aosta Valley, tick bites have been reported by Parini Hospital Service (personal communication [12]), however, information is lacking on the occurrence of ticks carrying zoonotic agents in this Alpine region, where occupational and recreational activities commonly take place. In this study, we collected questing ticks in three hiking trails in a municipality of Aosta Valley, and estimated the prevalence of infection by *Borrelia burgdorferi sensu lato* (s.l.) and *Rickettsia* spp.—the agents of tick—borne zoonoses, which are most frequently detected in *I. ricinus* in Europe [13]. Furthermore, we applied a risk assessment approach to estimate the probability of exposure of people to infected ticks, to provide suggestions to avoid tick bites, and to set the basis for further studies of a larger scale.

## 2. Materials and Methods

We adapted the terminology of the World Organisation for Animal Health (OIE) [14] to our case study: hazard characterization included the study of ticks and transmitted agents; release assessment was the estimation of the probability of finding infected, questing ticks in 100 m^2^ of land; exposure assessment was the estimation of the probability of people’s contact with infected ticks.

### 2.1. Hazard Characterization

#### 2.1.1. Ticks Collection

**Figure 1:**
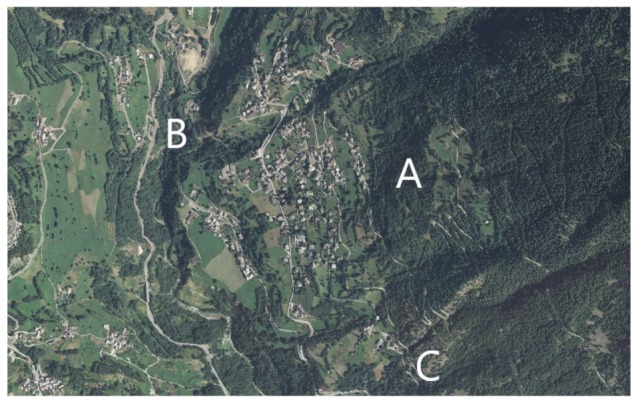
Aerial photograph of the inhabited center in Aosta Valley, where questing ticks were collected, from May through July, 2016. Letters indicate central locations of selected hiking trails.

Ticks were collected from May to July 2016 in a municipality in Aosta Valley, northwestern Italy (45°47′ N, 7°19′ E), where human tick bites had been reported [12]. We selected three hiking trails (A, B and C) neighboring the inhabited area, where recreational activities take place (Figure 1) [15]. Trail A was at an altitude ranging between 1037 and 1136 m a.s.l., and vegetation cover was mostly characterized by downy oak (*Quercus pubescens*). Trail B was at 785 m a.s.l., and vegetation included mixed wood (*Acer* spp., *Tilia* spp., *Fraxinus* spp.) and pasture. Conifer wood (*Pinus sylvestris*) dominated trail C (1025, 1050 m a.s.l.).

We collected questing ticks by dragging a 1 m^2^, white, cotton cloth on the ground vegetation, along 100 m^2^ transects, during several dragging sessions, on each trail, by stopping every 25 m to check the attachment of ticks on the drag and on the operators’ clothes (collection by walking) [16]. Every transect was divided into an internal part, with short vegetation, mostly constituted of leaf litter, and into an external part, with relatively tall vegetation, mostly constituted of grass, ranging ~20–50 cm, where we collected questing ticks separately.

Before performing the sampling, a data sheet was filled with Global Positioning System coordinates (Universal Transverse Mercator projection, zone 32N), temperature and humidity at each transect, by using a Samsung Galaxy smartphone (Samsung Electronics Italia, Milano, Italy) and a HI 8564 thermo hygrometer (Hanna Instrument Italia, Milano, Italy).

We collected nymphal and adult ticks only, which are the most important life stages for the transmission of zoonotic agents to people, especially *B. burgdorferi* s.l. [16]. Collected ticks were preserved in 70% ethanol, and subsequently identified under a microscope using taxonomic keys by Manilla (1998) [17].

#### 2.1.2. Molecular Analysis

We extracted a sample of 30 *I. ricinus* nymphs (10 from each of the three trails), and screened them by PCR to detect *B. burgdorferi* s.l. and *Rickettsia* spp. as described by Tomassone et al. (2017) [18]. Sample size was chosen so as to have 95% confidence of detecting bacterial agents in at least one tick, if prevalence of infected ticks was approximately 10% or greater. For the DNA extraction, we used DNeasy^®^ Blood & Tissue kit (Qiagen, Hilden, Germany). To identify *B. burgdorferi* s.l. we amplified the intergenic spacer region included between genes coding for the 5S and 23S subunits of ribosomal RNA. On the other hand, *Rickettsia* spp. infection was investigated using, first, a PCR targeting the *gltA* gene. Positive specimens were, subsequently, tested by another PCR, targeting the *ompA* gene, to characterize Spotted Fever group. Amplicons were purified using ExoSAP-IT PCR Clean-up Kit (GE Healthcare, Chalfont, UK) and sequenced at BNR Genomics (Padova, Italy). Sequences were analysed and edited by using DNASTAR Lasergene software (Madison, WI, USA), and submitted to BLAST (www.ncbi.nlm.nih.gov/BLAST) for comparison to reference sequences in GenBank.

### 2.2. Release Assessment

To assess the release of infected ticks by the environmental source, we estimated the probability (*R*) of collecting at least one infected nymph by dragging on a 100 m^2^ transect, by using the following equation [19]:

(1) $$R=1-e^{-PI\;\times \;DT}$$

where *PI* is the prevalence of *B. burgdorferi* s.l., or *Rickettsia* spp., as obtained by PCR; *DT* is the mean number of collected nymphs per 100 m^2^ dragging. *R* was separately calculated by using specific *DT* values from the three hiking trails and, discriminating between the internal and the external parts. The same *PI* value, on the other hand, was used in *R* calculation, by combining PCR results on ticks from all hiking trails.

### 2.3. Exposure Assessment

#### 2.3.1. Probability of Exposure (*E*)

The probability of exposure (*E*) can be defined as the probability of contact of a person with at least one infected tick along a 100 m^2^ trail. It was estimated by adapting Verheyen and Ruyts (2016) equation [20], which combines visitor flow (*v*), contact probability with questing nymphs (*c*), and release (*R*, Equation (1)).

(2) $$E=\left(v\;\times \;c\right)\times \;\left(1\;-\;e^{-PI\;\times \;DT}\right)$$

We defined *v* as the probability of at least one visitor per hour in a trail, based on observations, which were recorded during the sampling sessions; *v* was calculated by the following equation:

(3) $$v=1\;-\;e^{-VH}$$

where *VH* is the number of observed visitors per hour.

We defined *c* as the probability of contact between a visitor and questing nymphs; it was calculated as the ratio between the mean numbers of nymphs, which were collected by walking, divided by the mean numbers of nymphs, which were collected by dragging (*DT*). *E* was separately estimated for the three hiking trails (A, B and C), and for the trails’ internal, and external parts.

#### 2.3.2. Questionnaire

To integrate information on the exposure of people to questing ticks in the examined trails, we administered a short questionnaire to residents in the study area. The following questions were included: (1) number of people in the household; (2) number of people carrying out working or recreational activities in the specific hiking trails; (3) occurrence of tick bites on components of the households; and (4) geographic location of tick bites, to be identified on a municipality map. The questionnaires (*n* = 355) were manually delivered into mailboxes of each house of the municipality, asking to return the filled questionnaires in a box in the city hall. Questionnaires were anonymous, and data were only presented as frequency distributions of results of questions.

## 3. Results

### 3.1. Hazard Characterization

Dragging yielded 318 questing ticks in 9 transects (3 in trail A; 4 in trail B; 2 in trail C), including 285 nymphs and 31 adults *I. ricinus*, and 2 adults *D. marginatus*. Twenty *I. ricinus* were collected by walking (from collector’s clothes), of which, 11 nymphs, and 9 adults.

The mean number of *I. ricinus* nymphs, which were collected by dragging in 100 m^2^ transects, was 7.9 for the internal parts of trails, and 4.4 for the external parts. Considering the three trails separately, the mean number was 12.5 nymphs per 100 m^2^ dragging for trail A, 4.8 for trail B, and 3.5 for trail C. The mean number of adult *I. ricinus* was 0.44 in the internal parts of the trails, and 0.94 in the external parts. Trail A was characterized by relatively low mean temperature (23.1 °C) and high relative humidity (RH = 70.5%), in comparison with trails B (26.3 °C, 63.8% RH), and C (30.6 °C, 54.4% RH).

Prevalence of *B. burgdorferi* s.l. in *I. ricinus* nymphs (*PI*) 40.0% (95% CI: 22.5, 57.5), whereas prevalence of *Rickettsia* spp. was 13.3% (95% CI: 1.17–25.50). Sequence analysis showed the presence of *B. afzelii* in 10 out of 12 *B. burgdorferi* s.l.—positive nymphs (83.3%), and *B. valaisiana* was found in two nymphs (16.7%). *R. helvetica* was identified in one of the four *Rickettsia* spp.—positive *I. ricinus* nymphs.

### 3.2. Release Assessment

**Figure 2:**
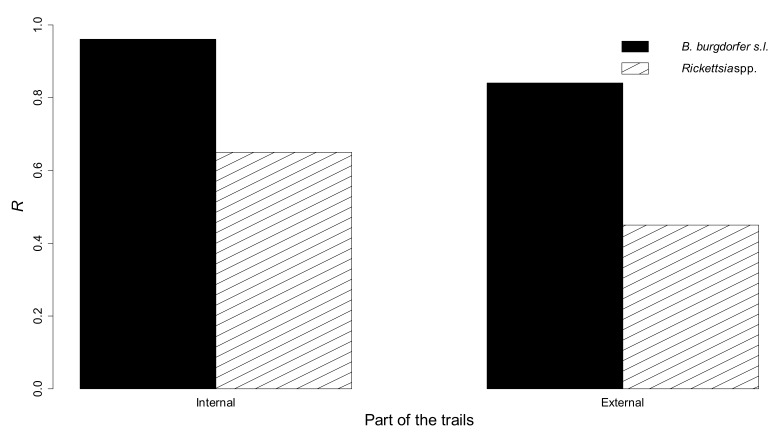
Probability (*R*) of collecting at least one infected, questing *I. ricinus* nymph, carrying *B. burgdorferi* s.l. or *Rickettsia* spp., by dragging in the internal and in the external parts (combined results) of three 100 m^2^ hiking trails in Aosta Valley.

**Figure 3:**
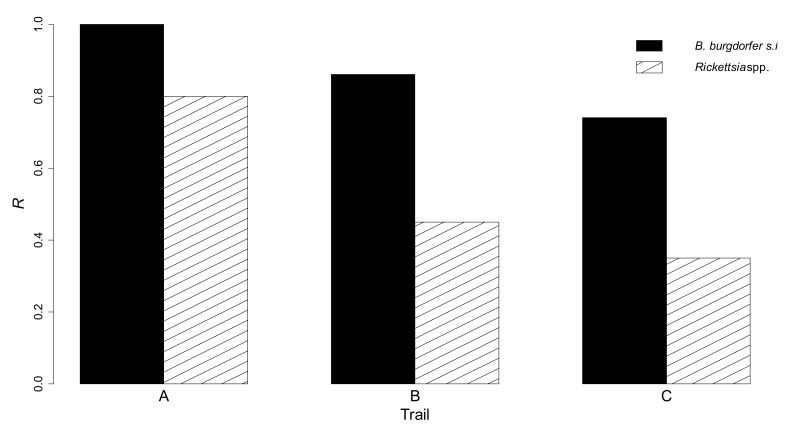
Probability (*R*) of collecting at least one infected, questing *I. ricinus* nymph, carrying *B. burgdorferi* s.l. or *Rickettsia* spp., by dragging in three hiking trails in Aosta Valley.

There was a trend for greatest *R* values in the internal part of all trails, and in trail A (Figure 2 and Figure 3).

### 3.3. Exposure Assessment

**Figure 4:**
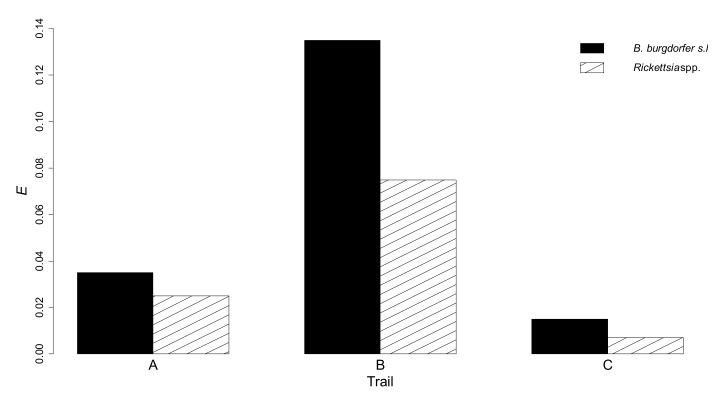
Probability (*E*) of exposure to questing ticks, carrying *B. burgdorferi* s.l. or *Rickettsia* spp., in the external parts of three hiking trails, in Aosta Valley.

*E*, as obtained by equation 2, was equal to 0 in the internal parts of trails, since no ticks were collected on the operator’s clothes, by walking on short vegetation. On the contrary, *E* indicated that exposure of people to infected nymphs was most likely in the external part of the trails and, particularly, in trail B (Table 1, Figure 4).

Sixty of the 355 questionnaires delivered were filled and returned. The resulting mean number of people per household was 2.7, and 89.1% of household members use the hiking trails for recreational reasons. Tick bites were reported by 46.8% of people during recreational activities. In ten out of 20 questionnaires including map locations, tick bites occurred on trail B, whereas only one tick bite was reported in trail A, and none in trail C. Other tick bites were reported in locations outside of the studied trails. No information on tick species and life stage was collected.

## 4. Discussion

*I. ricinus* was the most abundant tick species in the study area, in agreement with previous reports from other mountain areas in Italy, at ~1000 m a.s.l., and characterized by deciduous woods as the dominant vegetation cover [5], [10]. Dominance of downy oaks in trail A was associated with favourable habitat conditions for *I. ricinus*, characterised by high humidity, and likely abundance of hosts for ticks, such as rodents and ungulates. These factors might explain the greater numbers of questing nymphs collected in trail A, in comparison with other trails (Figure 3) [21,22].

The mean number of nymphs per 100 m^2^ collected by dragging in the internal part of trails (*DT* = 7.9) was greater than those reported in similar habitat types, in other Italian, northwestern regions (*DT* = 2.6, 3.5, in Piedmont [5]; *DT*= 0.16, 0.50 in Liguria [10]). Furthermore, the prevalence of *B. burgdorferi* s.l., which we detected in the small sample of nymphs tested (40.0%; 95% CI: 22.5, 57.5), was greater than prevalence in Piedmont (10.6%) [5]. Conversely, it was closer to the prevalence values in northeastern Italy [23,24]. Further studies, on a wider geographical scale, and on a greater sample of ticks, should be carried out, to investigate on common conditions, in these Alpine regions, underlying relatively great prevalence of infection.

The dominance of *B. afzelii* in infected *I. ricinus* nymphs suggests a major role of rodents (such as *Apodemus* spp.) as reservoirs for *B. burgdorferi* s.l. in the study area [25]. *B. afzelii* is a pathogenic genospecies, causing cutaneous disease. *B. valaisiana*, which was detected in two *I. ricinus* nymphs, is typically maintained by birds, and it is considered only potentially pathogenic [13].

Prevalence of *Rickettsia* spp. in *I. ricinus* nymphs (13.3%; 95% CI: 3.8, 30.7) was in agreement with the 3 to 14% prevalence range from other European studies [26]. *R. helvetica* is the only species which we identified by DNA sequencing. In the last few years, cases of rickettsiosis caused by *R. helvetica* have been reported in Europe [26]. The role of wild animals in the maintenance of *R. helvetica* is still uncertain, and it is hypothesized that birds might be competent reservoirs, since they may develop bacteraemia [27].

The greatest probability of encountering infected nymphs (*R*) in the internal part of hiking trails could have been affected by the method of collection. In fact, dragging is particularly suited for the collection of nymphs in short vegetation or on leaf litter [28]. Conversely, dragging is less effective to collect ticks in relatively tall vegetation, such as grass and shrubs, where ticks were most effectively collected by walking. Flagging is another method for sampling questing ticks, which might be more effective than dragging in the presence of grass and shrubs, especially for adult tick stages, and it could be included in further investigations [29]. In fact, the abundance of adult *I. ricinus* might have been underestimated in our study, even though this stage might be a source of exposure for people in relatively tall vegetation.

The probability of exposure of people to infected questing nymphs (*E*) in a trail was not directly correlated with *R*. In fact, *E* was greatest in the external part of trail B, both for *B. burgdorferi* s.l. (13.6%), and for *Rickettsia* spp. (7.4%). This was largely attributable to the greatest recorded frequentation of trail B by people (Table 1). *E* calculation was previously used by Verheyen and Ruyts (2016) [20] assuming *c* = 1.0 in vegetation taller than 50 cm, and *c* = 0.1 in shorter vegetation. Such an approximation could be used in the absence of information on tick collection by walking.

Results of the questionnaire on the frequentation of trails by people and on tick bites are in agreement with our estimate of a greatest *E* on trail B, although data on human activities on each trail, and in other locations where tick bites were reported, should be further investigated.

## 5. Conclusions

Walking in the internal part of hiking trails can be recommended, to reduce the probability of exposure to ticks and tick-borne zoonotic agents, even though this is in contrast with results from tick collection by dragging. Consequently, we suggest combining data on tick collection by both dragging and walking, to calculate *c*, and to use the probability of exposure model (*E*), rather than data from dragging alone, in risk assessment. The estimation of factor *v* (visitor flow) should be improved, by observing the passages of people, in each trail, for longer time periods. Indeed, although we demonstrated the occurrence of *I. ricinus*, and a relatively high prevalence of infection by *B. burgdorferi* s.l. and *Rickettsia* spp., the estimation of the probability of exposure of people to infected ticks cannot be generalized outside our limited study area. Our approach should be supported by more precise estimations of relevant parameters, including seasonal variations in tick activity and of *E*. Further studies on the spatial and temporal patterns of tick activity in Aosta Valley are then recommended, which could serve as the basis for proper information, and for the prevention of the exposure of people to infected ticks.

## Figures and Tables

**Table 1:** Calculation of the probability of exposure (*E*) of people to questing ticks, carrying *B. burgdorferi* s.l. or *Rickettsia* spp., in the external part of three hiking trails, in Aosta Valley.

Trail	*DT_walking_*	*DT*	*c*	*VH*	*v*	*R*		*E*	
						Bb	Rick	Bb	Rick
A	0.75	10.5	0.07	0.5	0.39	0.99	0.80	0.035	0.028
B	0.2	2.6	0.08	2	0.86	0.85	0.46	0.136	0.074
C	0.25	3	0.08	0.25	0.22	0.75	0.37	0.015	0.007

*DT*_walking_ = mean number of nymphs collected from operators’ clothes; *DT* = mean number of nymphs collected by dragging; *c* = ratio between *DT*_walking_ and *DT*_dragging_; *VH* = number of visitors per hour; *v* = probability of at least one visitor per hour (equation 3); *R* = probability of collecting at least one infected nymph by dragging on a 100 m^2^ transect; Bb = *B. burgdorferi* s.l.; Rick = *Rickettsia* spp.; *E* = probability of exposure to ticks carrying *B. burgdorferi* s.l. or *Rickettsia* spp.
